# Selective Removal of Only Clinically Suspicious Positive Lymph Nodes Instead of a Complete Inguino-Femoral Lymph Node Dissection in Squamous Cell Carcinoma of the Vulva

**DOI:** 10.3390/cancers15153844

**Published:** 2023-07-28

**Authors:** Guus Fons, Nikki B. Thuijs, Ming Tjiong, Lukas J. A. Stalpers, Jacobus van der Velden

**Affiliations:** 1 Department of Gynecologic Oncology, Amsterdam UMC, University of Amsterdam, Meibergdreef 9, 1105 AZ Amsterdam, The Netherlands; g.fons@amsterdamumc.nl (G.F.); m.tjiong@amsterdamumc.nl (M.T.); 2 Department of Pathology, Amsterdam UMC, University of Amsterdam, Meibergdreef 9, 1081 HV Amsterdam, The Netherlands; n.thuijs@amsterdamumc.nl; 3 Department of Radiotherapy, Amsterdam UMC, University of Amsterdam, Meibergdreef 9, 1105 AZ Amsterdam, The Netherlands; l.stalpers@amsterdamumc.nl

**Keywords:** vulvar cancer, bulky lymph nodes, lymph node debulking

## Abstract

**Simple Summary:**

When there is evidence for the spread of a vulvar tumor to the regional lymph nodes in the groins, local resection of the primary tumor and complete removal of these regional lymph nodes, which is frequently followed by radiotherapy, is recommended. This treatment results in significant side effects. Alternative treatment methods with fewer side effects are therefore being explored. In this study, 40 patients with evidence for spread of the tumor to the lymph nodes, resulting in large (bulky) nodes, were treated with removal of the enlarged lymph nodes only, followed by radiotherapy, leaving behind the nodes that looked normal. The survival of this group was compared with a similar group of 37 patients treated with the removal of all nodes in the groin(s). Although in both groups the survival rate was poor (5 years survival: 29.4% vs. 23.8%), there was no difference between the groups. Therefore, we recommend removing only bulky nodes in cases where there is a proven spread of the tumor in these nodes.

**Abstract:**

Background. The oncological safety of only removing bulky, positive groin lymph nodes followed by radiotherapy without performing a complete inguino-femoral node dissection (IFL) in squamous cell cancer of the vulva is based on two small studies. The aim of this study was to confirm the oncological safety of this treatment policy. Methods. The survival of consecutive patients with clinically suspicious and pathologically positive groin nodes treated with the selective removal of these nodes followed by radiotherapy was compared with the survival in historical controls matched for the variables extranodal spread and diameter of the metastasis > 15 mm and treated with a complete IFL. Results. There was no difference in disease-specific survival between patients treated with debulking (*n* = 40) versus complete IFL (*n* = 37) (43.1% vs. 44.8%, *p* = 0.336, respectively). Overall, survival and groin recurrence-free survival did not differ between the groups either. Conclusion. This retrospective study in a cohort of women with vulvar cancer corroborates previous smaller studies that have shown that the selective removal of suspicious inguinal nodes yields similar oncological outcomes compared with patients matched for important prognostic variables and treated with a complete IFL when both are followed by radiotherapy.

## 1. Introduction

Vulvar cancer is a rare disease, and it mainly affects elderly women [1]. Metastases in the inguinal femoral lymph nodes occur frequently and are related to pathological variables such as depth of invasion, lymph vascular space invasion, and tumor size [2]. The standard treatment for patients with squamous cell cancer of the vulva and a depth of invasion > 1 mm consists of a radical local excision of the primary tumor and either lymph node evaluation by sentinel node dissection (SLN) or primary inguinal femoral lymph node dissection (IFL) [3]. Adjuvant radiotherapy is recommended in patients with metastases in the nodes, with the exception of patients with a single clinically occult intracapsular metastasis [4].

Theoretically, there are several treatment strategies when a patient, pre- or perioperative, presents with pathologically confirmed positive lymph nodes in the groin. Other than the previously mentioned standard treatment, resection of only the tumor-positive lymph node(s) followed by radiotherapy is an alternative for the standard IFL. This positive lymph node could be a positive SLN or any other pre- or perioperative detected positive lymph node. Finally, there is the option of primary radiotherapy, without surgery, in patients with a cyto- or histopathological confirmed tumor-positive lymph node. The aim of all these strategies is to decrease the considerable morbidity following a complete IFL and adjuvant radiotherapy without compromising survival [5].

Unfortunately, for many of these strategies, there is no sound scientific evidence regarding their efficacy or oncological safety. Only one prospective study that was published recently has been performed in this context (GROINSS-VII) [6]. With a groin recurrence rate of 1.6%, this study showed that it is safe to treat a patient with only radiotherapy for the groin(s) in the case of a single positive SLN with a diameter of the intra nodal metastasis ≤ 2 mm. Patients not fulfilling these criteria showed a higher (20%) than expected groin recurrence rate after radiotherapy only. Therefore, a complete IFL is recommended for this latter group of patients.

Only two small retrospective cohort studies on the selective removal of bulky positive lymph nodes followed by radiotherapy have been published [7,8]. These studies included patients with “clinically suspicious and histologically confirmed positive nodes” [7] and “clinically involved or enlarged lymph nodes” [8]. Both studies showed that tumor control in the groin and/or survival were not different from patients treated with a complete IFL. The latter study also showed that the complication risk was lower after debulking of the nodes compared with a complete IFL [8]. Despite an apparent advantage for debulking of the nodes, the guidelines of the European Society of Gynaecological Oncology (ESGO) state that there is no preference for either of the two procedures, e.g., debulking or complete IFL [3].

After our publication in 2007 [7], we changed our surgical treatment protocol for patients with bulky groin nodes from complete IFL to only debulking of the nodes, both followed by radiotherapy. The aim of this retrospective single center cohort study was to compare the oncological outcome of debulking suspicious groin nodes with historical controls, matched for important prognostic variables, and treated with a complete IFL, with the hypothesis that the oncological outcome of both treatment strategies did not differ.

## 2. Materials and Methods

### 2.1. Patients and Treatment

The database of the Department of Gynecologic Oncology of the Amsterdam UMC was examined to identify patients, retrospectively, with squamous cell cancer of the vulva (SCC) and histologically confirm positive groin lymph nodes, treated between 1985 and 2020 in a curative setting. Local treatment consisted of either a radical vulvectomy (before 1995), local radical excision (after 1995), or primary chemo-radiotherapy. Treatment of the lymph nodes before 1995 was carried out bilaterally through en bloc resection together with the radical vulvectomy. After 1995, either a unilateral or bilateral IFL was carried out through separate incisions. After 2007, preoperative or perioperative enlarged and histologically confirmed positive groin nodes were treated with lymph node debulking. Debulking was performed through a linear incision 1 cm above and parallel to the groin crease. All palpably enlarged nodes were removed and (if not performed pre-operatively) a confirmation of tumor positivity was obtained by frozen section analysis. From 2001, a sentinel node procedure was carried out in patients with a unifocal tumor and a diameter < 4 cm in the absence of clinically suspicious groin nodes. Whenever a positive SLN was found, patients were treated with a complete (bilateral) IFL. In 2006, in the context of the GROINSS-VII study [6], patients with a single intranodal metastasis were treated with radiotherapy for the groin. After amendment to the protocol in 2010, only patients with a single intranodal metastasis ≤ 2 mm were treated with further groin radiotherapy, while all other patients underwent a complete IFL.

After a complete IFL, patients with >1 positive lymph node or extranodal spread received adjuvant radiotherapy. Patients with primary chemo-radiotherapy who had a lymph node dissection after the radiotherapy were excluded. After 2003, radiotherapy was CT-scan-guided. In principle, the fields incorporate the inguino-femoral nodal groups and the pelvic nodes below the sacroiliac joints. Although there was substantial individual variation, in general, the patients received 54 Gy in 2.0 Gy fractions for intra-nodal metastases and 60 Gy whenever extranodal disease was present. Due to the individualization of management plans, however, the radiation field was not consistent between patients. In some cases, this meant bilateral inguino-femoral radiotherapy; in others with negative contralateral nodes, unilateral fields were used. Some patients’ fields incorporated the vulva; others did not, while some patients received concurrent chemotherapy. The following data were retrieved from the database, patient files, and pathology reports: age, 2009 FIGO and cTNM stage, cell type, clinical and pathological tumor size, type of surgery regarding the inguino-femoral lymph nodes, number and laterality of tumor-positive lymph nodes, largest diameter of a metastasis in a tumor-positive lymph node, extra capsular spread of the lymph node, type of adjuvant treatment, and follow-up status. Missing pathological data were retrospectively reviewed by a dedicated resident pathologist (NT). After treatment, patients were seen for follow-up every 3 months during the first two years, every 6 months during the third year, and once yearly thereafter, depending on the presence of residual pre-invasive vulvar disease. Routine follow-up included history and physical examination. Recurrences were stratified as “local”, defined as a recurrence on the vulva; “regional”, defined as a recurrence in the groins; and “distant”, defined as any other recurrence. Combined recurrences were defined as a combination of loco-regional recurrence and distant recurrence. To compare the oncological outcome between the group of patients who were treated with debulking of the groin nodes versus a complete IFL, patients were matched for the variables extranodal spread and diameter of the metastasis > 15 mm.

### 2.2. Statistical Analysis

The main aim of this study was to analyze the recurrence rate/localization and survival after the removal of bulky lymph nodes only, as compared with matched controls with a complete IFL, both followed by radiotherapy. An independent sample t test was used to determine differences between the median age. To determine significant differences in distribution of clinical or pathologic variables among groups, a Fisher exact test was used. Survival was determined using the Kaplan–Meier method. Disease-specific survival (DSS) was defined as survival corrected for death from causes other than vulvar cancer. The groin recurrence-free survival was defined as the time from initial surgery to the date of first groin recurrence. A Cox proportional hazard model was used to compare disease-specific survival in patients who were treated with debulking versus a complete IFL, including the co variates age (≤74 vs. >74 years), time period of treatment (1984–1994; 1995–2005; 2006–2015; 2016–2020), extra capsular spread (yes vs. no), size of the metastases (≤15 mm vs. >15 mm), and 2009 FIGO stage. A *p*-value of <0.05 was considered statistically significant for all analyses. The analysis was performed on a per patient basis.

## 3. Results

From the database, 241 consecutive patients with SCC of the vulva and positive inguino-femoral lymph nodes treated with curative intent were identified.

The group of patients who were treated with debulking were matched for the prognostic variables extracapsular spread and size of the metastasis in the lymph node (>15 mm) with the total group of patients who had an IFL. As shown in Table 1, there were no differences in the frequencies of clinical and pathological characteristics in the group who were treated with nodal debulking (*n* = 40) compared with the group who had an IFL (*n* = 37). All stage IVB patients were assigned stage IVB because of histologically proven positive pelvic lymph nodes without distant metastases and were treated in a curative setting.

**Figure 1:**
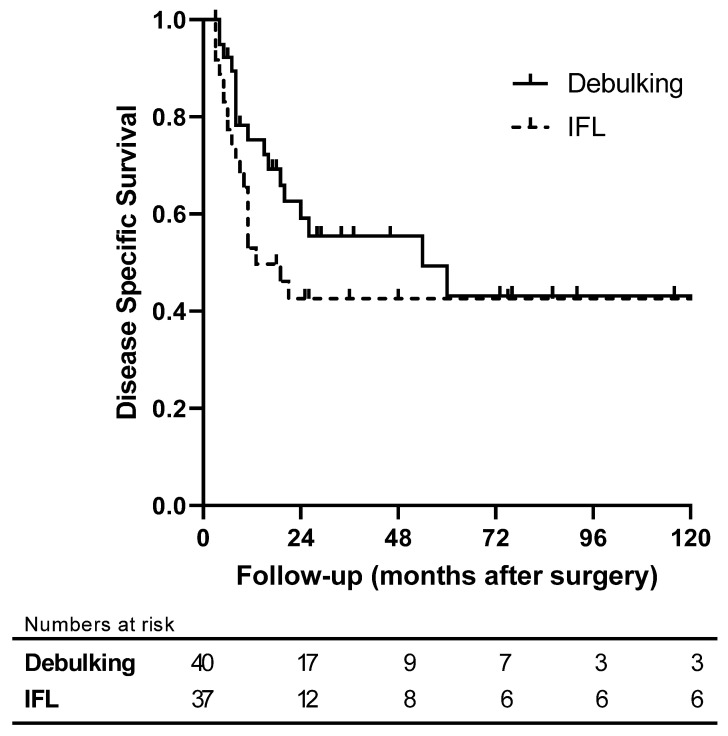
Comparison of disease-specific survival of patients who were treated with nodal bulking compared with matched controls (matched for extracapsular spread and size of the metastasis in the node) who were treated with an inguino-femoral lymph node dissection.

The median follow-up of censored patients was 31.5 months (range: 3–395). In this matched cohort, the actuarial DSS for patients who had nodal debulking (*n* = 40) did not differ from the patients who had an IFL (*n* = 37) (5-year DSS: 43.1% vs. 41.4%, *p* = 0.217). (Figure 1).

In the multivariate analyses, only the FIGO stage was an independent predictive factor for DSS (*p* < 0.008), while the type of treatment (debulking versus complete IFL) was not predictive (*p* = 0.136) (Table 2).

The 5-year groin recurrence-free and overall survival of the debulking group versus the IFL group did not differ either (respectively, 80.6% vs. 79.4%, *p* = 0.421, and 29.4% vs. 23.8%, *p* = 0.286).

The recurrence pattern for the two treatment groups is shown in Table 3. Both in the IFL group and debulking group, recurrences with a distant component were predominant (17/22 (77%) and 17/21 (81%), respectively). No isolated groin recurrences occurred.

## 4. Discussion

### 4.1. Summary of Main Results

In this retrospective study, we found that the oncological outcome, both in terms of survival and groin recurrence rate of patients with clinically suspicious positive groin lymph nodes, treated with a lymph node debulking followed by radiotherapy, was not compromised compared with patients who had a complete IFL matched for the variables extranodal spread and diameter of the metastasis > 15 mm. Recurrences with a distant component were predominant in both groups.

### 4.2. Results in the Context of the Published Literature

The outcome of the current study is grossly in line with a previous, smaller study, where patients with bulky nodes treated in our center by a complete IFL (*n* = 23) were compared with patients from two Australian gynecologic oncology centers, treated with lymph node debulking (*n* = 17) [7]. The groin recurrence-free survival did not differ between both groups, while disease-specific survival was better in the nodal debulking group. There is only one other study that addresses the same issue, in which it was concluded that patients with clinically suspicious nodes and/or macro metastases > 2 mm showed no difference in groin recurrence-free survival after nodal debulking, followed by radiotherapy, compared with a complete IFL, while treatment-associated morbidity was significantly lower in the group of patients treated with debulking. Based on the findings in the latter study, the recommendation was that nodal debulking followed by radiotherapy was the preferred mode of treatment in cases where clinically suspicious nodes and/or macro metastases > 2 mm were found [8]. It is, however, questionable if this recommendation is valid regarding a group of patients with any metastasis > 2 mm, because in the recently published results of the GROINSS-VII study, patients with a sentinel node metastasis > 2 mm (all clinically non-suspicious nodes), treated with radiotherapy, showed a groin recurrence rate at 2 years of 22.0% versus 6.9% in patients who underwent IFL, with or without adjuvant radiotherapy [6]. Therefore, we must await the results of the ongoing GROINSS-VIII study, analyzing the oncological safety of concurrent chemotherapy and radiotherapy for the group of patients with a metastasis > 2 mm in the sentinel node [9].

An explanation for the fact that in the current and previous studies, no survival difference was found between debulking bulky nodes versus the more radical complete IFL, both followed by radiotherapy, could be the very high risk of distant metastases (80%) and the resultant death of disease in this group of patients with bulky nodes. In that context, a more radical loco regional treatment will, most likely, have no impact on survival.

The role of primary (chemo) radiotherapy for patients with bulky nodes in the groin has not been extensively studied. We could find only one study reporting on the results of definitive primary (chemo)radiotherapy in 33 patients with “gross inguinal lymphadenopathy” [10]. Patients were included if they had histologic evidence (*n* = 8), and/or strong clinical evidence (*n* = 29 palpable nodes), and/or radiographic evidence (*n* = 33) (median long axis 2.5 cm range 1.4–8.7 cm) of regional metastasis. Two patients were treated with debulking of a positive node. The radiotherapy dose given was 66 Gy (60–70 Gy), depending on the size of the lymph node. Twenty patients also had concurrent chemotherapy. Four of thirty-three (12%) patients had recurrent or progressive groin disease, and the 3-year overall survival rate was 51%. Although the 12% groin recurrence rate seems comparable to our results (11% groin recurrence rate), the patient populations of the two studies cannot be compared because histologic confirmation of positive lymph nodes was only performed in 8/33 (24%) patients in the latter study [10], while this was 100% in the current study. Positive predictive values of 62% for CT scan [11], 52% for PET CT scan [12], 65% for ultrasound [13], and 46% for MRI [14] were reported in vulvar cancer. It is clear from these data that suspicious nodes on imaging without pathological confirmation, which was the selection criterion for the majority of patients in the study by Stecklein et al. [10], carry the risk of overestimating the presence of lymph node metastases in the groins. Therefore, this procedure can potentially lead to false conclusions regarding the efficacy of definitive radiotherapy for the groins.

### 4.3. Strength and Weaknesses

A strength of our study is the fact that we could compare the oncological outcome of debulking versus IFL after matching for the most important prognostic variables, such as extranodal spread and size of the metastasis in the node. Although we might argue that a relative strength of our study is the fact that this is the largest study on the subject of the debulking of groin nodes so far, at the same time, we realize that the numbers in this retrospective study are still small (40 vs. 37), which is a significant weakness. A formal non-inferiority prospective study with large numbers, however, seems unrealistic because of the low incidence of vulvar cancer, particularly for this subgroup of patients with bulky lymph nodes. A weakness could also be the fact that we used historical controls, sometimes being treated > 30 years ago. Debulking was the treatment of choice in later years, while complete IFL was the treatment of choice mainly in the earlier years of the study (as shown in Table 1). The introduction of pre-treatment imaging and changes in radiotherapy treatment over the years could potentially lead to prognostically different groups. However, by matching the historical controls for the most important prognostic variables, extranodal spread and diameter of the metastasis, this potential selection bias can be diminished.

### 4.4. Implications for Practice and Future Research

It is interesting to see that recommendations in various national guidelines regarding the policy for patients with bulky nodes either differ or are not mentioned at all (Table 4).

Both in the BGCS [15] and JSGO [16] guidelines, the recommendation is to selectively remove bulky lymph nodes before (chemo) radiotherapy is administered. In the NCCN guideline, it is recommended to tailor treatment, without specifically defining what “tailoring” means [17]. In the DGGG [18] and the GOC [19] guidelines, no specific recommendation is given for patients with bulky nodes, while the ESGO guideline [3] states that the optimal management has to be defined. Unfortunately, there is also no agreement on the strict definition of a “bulky” lymph node. The studies analyzing either debulking bulky nodes or primary radiotherapy for bulky nodes use the terminology “clinically suspicious” [7,8] or “gross inguinal lymphadenopathy” [10] without mentioning a specific size of the suspicious node. Our data show that in the debulking group, the median clinically measured size of the “bulky” node was 29 mm (range: 14–70). However, both from our current data and from the collated literature data, there is no scientific evidence to recommend a specific size in relation to the definition of bulky node.

### 4.5. Conclusions

Based on the current study, we feel that patients with clinically suspicious bulky lymph nodes after the removal of the bulky nodes do not need a further complete IFL but can be treated with additional inguino-femoral radiotherapy. Primary (chemo) radiotherapy for bulky nodes could be an alternative treatment, although more studies are needed in patients where pathological/cytological confirmation of the suspicious nodes is mandatory.

## Figures and Tables

**Table 1:** Clinical and pathological characteristics of patients who were treated with a nodal debulking compared with matched controls (for extracapsular spread and size of the metastasis in the node) who were treated with an inguino-femoral lymph node dissection.

Variable	Debulking (*n* = 40)	IFL (*n* = 37)	*p* Value
Med Age (Range)	74 (36–90)	77 (34–92)	0.364
Treatment period			
• 1984–1994	• 0 (0%)	• 14 (38%)	
• 1995–2005	• 3 (8%)	• 18 (49%)	
• 2006–2016	• 20 (50%)	• 3 (8%)	
• 2017–2020	• 17 (42%)	• 2 (5%)	0.001
FIGO (2009) stage			
• IIIA	• 6 (15%)	• 4 (11%)	
• IIIB	• 8 (20%)	• 7 (18%)	
• IIIC	• 20 (50%)	• 21 (57%)	
• IVA	• 2 (5%)	• 0 (0%)	
• IVB	• 4 (10%)	• 5 (14%)	0.647
Med tumor diameter in mm (range)	46 (10–150)	48 (20–90)	0.612
Clinical ^1^ size of suspicious node in mm (med/range)	29 (14–70)	21 (11–70) ^2^	0.245
Extra capsular spread	25/40 (63%)	25/37 (68%)	0.811
Diameter nodal metastasis > 15 mm	32/40 (80%)	29/37 (78%)	1.000

^1^ Clinical size was determined either by palpation, imaging, or both; ^2^ *n* = 5 missing.

**Table 2:** Multivariable Cox model for disease-specific survival analysis in the study population with two treatment categories (debulking versus complete IFL).

Covariates	HR	95% CI	*p*-Value
Debulking vs. complete IFL	0.45	0.15–1.29	0.14
FIGO stage	1.63	1.13–2.34	0.008
Extranodal spread (yes/no)	1.67	0.63–4.40	0.30
Size metastasis (> vs. ≤15 mm)	1.35	0.43–4.20	0.61
Age group (> vs. ≤74)	1.25	0.64–2.42	0.51
Time period of treatment	1.14	0.70–1.86	0.59

**Table 3:** Recurrence localization of the group who had an IFL compared with the group who were treated with debulking, both followed by radiotherapy.

	Isolated Local	Isolated Pelvis	Isolated Distant	Combination ^1^	Total
IFL	1	4	7	10	22
Debulking	4	0	7	10	21
Total	5	4	14	20	43

^1^ Combination included recurrence localizations local and/or groin and/or pelvis with distant.

**Table 4:** A selection of recommendations in various (inter)national guidelines regarding treatment policies in cases where bulky lymph nodes are found.

	Guidelines Regarding the Treatment of Bulky Lymph Nodes in the Groins
ESGO ^1^ 2016 [3]	The optimal management of the groin (full inguino-femoral lymphadenectomy or isolated removal only) for enlarged, proven metastatic nodes remains to be defined.
BGCS ^2^ 2020 [15]	In an effort to reduce complications from dual modality treatment, lymph node debulking rather than formal lymphadenectomy may be used prior to (chemo) radiotherapy.
JSGO ^3^ 2018 [16]	It seems likely that at least the excision of swollen lymph nodes suspected of metastasis should be considered, and the presence/absence of metastasis examined histologically, before considering radiation therapy.
NCCN ^4^ 2022 [17]	Any nodes that are grossly enlarged or suspicious for metastases during the unilateral inguino-femoral lymphadenectomy should be evaluated by frozen section pathology intraoperatively in order to tailor the extent and laterality of the lymphadenectomy.
DGGG ^5^ 2015 [18]	Systematic inguino-femoral lymphadenectomy (= surgical staging of the inguinal region) must always include removal of both the superficial (inguinal) and the deep (femoral) lymph nodes below the cribriform fascia (expert consensus).
GOC ^6^ 2019 [19]	Lymph node assessment can be performed by either complete IFLD or sentinel lymph node mapping, depending on tumour size and the presence or absence of clinically enlarged lymph nodes.

^1^: ESGO = European Society of Gynecologic Oncology; ^2^: BGCS = British Gynecological Cancer Society; ^3^: JSGO = Japanese Society for Gynecologic Oncology; ^4^: NCCN = National Comprehensive Cancer Network; ^5^: DGGG = Deutsche Gesellschaft fur Gynakologie und Geburtshilfe; ^6^: GOC: Society of Gynecologic Oncology of Canada.

## Data Availability

In accordance with the journal’s recommendations, we will provide our data for independent analysis for the purposes of additional data analysis or for the reproducibility of this study in other centers if such is requested.
